# Atrial Natriuretic Peptide_31–67_: A Novel Therapeutic Factor for Cardiovascular Diseases

**DOI:** 10.3389/fphys.2021.691407

**Published:** 2021-07-08

**Authors:** Gustavo Jose Justo da Silva, Raffaele Altara, George W. Booz, Alessandro Cataliotti

**Affiliations:** ^1^Institute for Experimental Medical Research, Oslo University Hospital and University of Oslo, Oslo, Norway; ^2^Department of Pathology, School of Medicine, University of Mississippi Medical Center Jackson, Jackson, MS, United States; ^3^Department of Pharmacology and Toxicology, School of Medicine, The University of Mississippi Medical Center, Jackson, MS, United States

**Keywords:** natriuretic peptides, atrial natriuretic peptide, proANP_31–67_, cardiovascular disease, heart Failure

## Abstract

The characterization of the cardiac hormone atrial natriuretic peptide (ANP_9__9__–__1__26_), synthesized and secreted predominantly by atrial myocytes under stimulation by mechanical stretch, has established the heart as an endocrine organ with potent natriuretic, diuretic, and vasodilating actions. Three additional distinct polypeptides resulting from proteolytic cleavage of proANP have been identified in the circulation in humans. The mid-sequence proANP fragment 31–67 (also known as proANP_3__1__–__6__7_) has unique potent and prolonged diuretic and natriuretic properties. In this review, we report the main effects of this circulating hormone in different tissues and organs, and its mechanisms of actions. We further highlight recent evidence on the cardiorenal protective actions of chronic supplementation of synthetic proANP_3__1__–__6__7_ in preclinical models of cardiorenal disease. Finally, we evaluate the use of proANP_3__1__–__6__7_ as a new therapeutic strategy to repair end-organ damage secondary to hypertension, diabetes mellitus, renal diseases, obesity, heart failure, and other morbidities that can lead to impaired cardiac function and structure.

## Introduction

### Natriuretic Peptides

**FIGURE 1:**
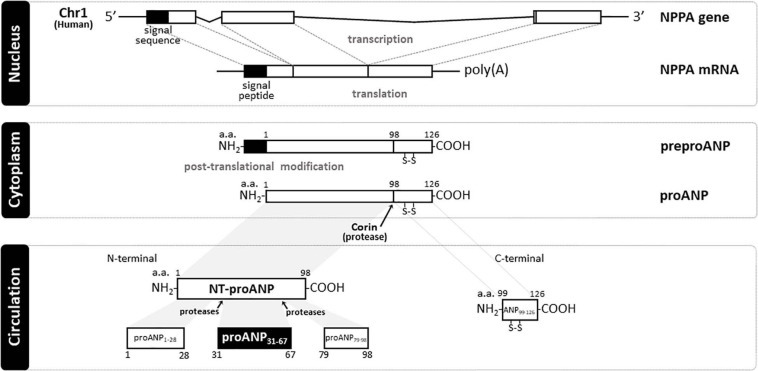
Schematic representation of natriuretic peptide gene product processing and generated peptide fragments. In humans, atrial natriuretic peptide (ANP) is encoded by NPPA gene (Chr1:11,845,709-11,848,345:-) that translates a 151 a.a. polypeptide (preproANP). Post-translational modification process cleavages the 25 a.a. signal sequence to produce proANP, a 126 a.a. peptide that is stored in intracellular granules of atrial myocytes. Under stimulation, atrial cells release proANP that is rapidly converted to the 28-a.a. C-terminal mature ANP by the transmembrane serine protease Corin, and a 98 a.a. N-terminal mature ANP (NT-proANP or ANP_1__–__9__8_). Further cleavage by proteases of NT-proANP generates different fragments, i.e., amino acids 1–28 (proANP_*l*__–__28_), amino acids 31–67 (proANP_3__1__–__6__7_), and amino acids 79–98 (proANP_7__8__–__9__8_). NH2, free amine group located at the N-terminal end of a polypeptide; COOH, free carboxyl group located at the C-terminal end of a polypeptide; poly(A), multiple adenosine monophosphates mRNA tail; a.a., amino acid; Chr 1, Chromosome 1; NPPA, human atrial natriuretic peptide gene; ANP, Atrial natriuretic peptide; NT-proANP, N-terminal proatrial natriuretic peptide; preproANP, precursors to prohormone of atrial natriuretic peptide; proANP, prohormone of atrial natriuretic peptide.

The human natriuretic peptides (NPs) consist of a family of three known peptides encoded in the human genome, with each being a distinct gene product with similar structure (Figure 1). The atrial natriuretic peptide (ANP_9__9__–__1__26_), a hormone synthesized and secreted predominantly by cardiac cells, was the first member of the NP family to be discovered in de Bold et al. (1981), and established the heart as an endocrine organ. Wall stretch, due to increased intravascular volume and/or cardiac transmural pressure, is the major stimulus for cardiac ANP release (Goetze et al., 2020). ANP is encoded by the NPPA gene located on chromosome 1 in the human genome, and is primarily expressed by atrial myocytes. The NPPA gene translates a 151-amino acid polypeptide known as preproANP. A post-translational modification process cleaves the 25 amino acid signal sequence to produce proANP, a 126 amino acid peptide that is stored in intracellular granules of atrial myocytes (Yan et al., 2000). Under stimulation, atrial cells release proANP that is rapidly converted to the 28-amino-acid C-terminal mature ANP by the transmembrane serine protease corin (Yan et al., 2000), a transmembrane cardiac serine protease, to form the biologically active carboxyl-terminal 28-amino-acid peptide called ANP_9__9__–__1__26_ (Forssmann et al., 1998). The 28-amino acid peptide contains a 17-amino acid ring in the center of the molecule (Figure 1), formed by a disulfide bond between two cysteine residues at positions 7 and 23. The highly biologically active ANP_9__9__–__1__26_ is formed at equimolar amounts as the biologically inactive amino-terminal portion (98 amino acid) of proANP (termed NT-proANP, or proANP_1__–__9__8_) (Yan et al., 2000). However, as ANP_99__–__126_ has a very short half-life (less than 5 min) compared with NT-proANP (60–120 min), NT-proANP is considered a more reliable biomarker than ANP_9__9__–__1__26_ (Buckley et al., 1999). Originally, the ring structure was thought to be essential for the ANP_9__9__–__1__26_ biological actions (Currie et al., 1984), but linear forms of the N-terminal ANP prohormone containing internal sequences believed to account for activity without the ring structure were shown also to have biological activity, albeit significantly reduced (Brenner et al., 1990; Vesely, 2007). The B-type natriuretic peptide (BNP), also known as brain natriuretic peptide, is a hormone secreted primarily by cardiomyocytes in the heart atria and ventricles (Sudoh et al., 1988) in response to stretching caused by increased ventricular blood volume and increased filling pressure (de Lemos et al., 2003). While the main source of BNP in normal conditions is the atrium, the production of BNP from the ventricles increases under pathological conditions such as cardiac remodeling (Luchner et al., 1998). Under stimulation, a 32-amino acid polypeptide is secreted attached to a 76-amino acid N-terminal fragment in the prohormone called NT-proBNP. A specific convertase (furin or corin) subsequently cleaves proBNP between arginine-102 and serine-103 into NT-proBNP and the biologically active 32-amino acid polypeptide BNP 1–32. Last, the C-type natriuretic peptide (CNP), encoded by the gene NPPC located on human chromosome 2, is synthesized and secreted from the central nervous system (e.g., cerebellum, hypothalamus, and anterior pituitary), kidney, and vascular endothelial cells (Mukoyama et al., 1991; Heublein et al., 1992; Stingo et al., 1992; Suga et al., 1992), and by the heart (Vollmar et al., 1993; Del Ry et al., 2011; Sangaralingham et al., 2020), in response to shear stress and certain proinflammatory cytokines. CNP is structurally related to ANP and BNP molecules, but has less intensive natriuretic and diuretic effects (Goetze et al., 2020). A recent study revealed that CNP regulates distal arteriolar and capillary blood flow via NPR-B-induced cGMP signaling in microvascular smooth muscle cells and pericytes (Spiranec et al., 2018), controlling microvascular resistance and blood pressure through vasodilating actions (Sangaralingham and Burnett, 2018). Other NPs have been identified in nature. The *Dendroaspis* natriuretic peptide (DNP), is structurally similar to ANP, BNP, and CNP, and possesses comparable biologic properties to other NPs. Additionally, the NP urodilatin (URO or CDD/ANP 95–126), known as renal ANP among the NPs, is secreted by cells of the distal tubule and collecting duct in the kidney in response to increased blood pressure and blood volume. Urodilatin is transcribed by the NPPA gene, but differentially processed in the kidney, and only detected in urine (Meyer et al., 1998). Both DNP and Urodilatin bind to NPR-A resulting in a cGMP-dependent signal transduction (Pandey, 2014).

Biological actions of NPs are mediated by membrane-bound guanylyl cyclase receptors, which are expressed in a variety of cells. Three NP receptors are known: NPR-A (or NPR1), NPR-B (or NPR2), and NPR-C (or NPR3). ANP and BNP bind primarily to NPR-A and CNP binds to NPR-B (Charles et al., 1996; Kuhn, 2004). When activated, NPR-A and NPR-B receptors generate the second messenger cyclic guanosine monophosphate (cGMP), while the activation of NPR-C does not generate cGMP. In cardiac myocytes, cGMP-mediated signaling is regulated in a spatial and temporal manner by specific phosphodiesterases, which act to localize and temper levels of this signaling second messenger (Dunkerly-Eyring and Kass, 2020). All three NPs are ligands with similar affinity (Anand-Srivastava and Trachte, 1993) to the receptor NPR-C, which is not a guanylyl cyclase-linked receptor (Kuhn, 2004). It is known that NPR-C couples to inhibitory G proteins (G_i_) and causes inhibition of adenylyl cyclase and activation of phospholipase-C (Anand-Srivastava, 2005).

### Physiological Actions of the Linear Fragment ANP_3__1__–__6__7_

A comprehensive biological understanding of NPs emerged following studies in cultured cells, rodent models of altered NPs production or receptor function, and integrative physiologic studies in disease models and in humans. The biological properties of the NPs, which include natriuresis, vasodilatation, inhibition of the renin-angiotensin-aldosterone system (RAAS), positive lusitropy, and inhibition of fibrosis, have led to the unique concept of cardiorenal protection by activation of cGMP (de Bold et al., 1981; Burnett et al., 1984; Wada et al., 1994; Kishimoto et al., 1996; Stevens et al., 1996; Wright et al., 1996; Lainchbury et al., 2000). An accumulating body of evidence demonstrated the tissue-specific distribution of NPs (Saito et al., 1989; Kojima et al., 1990; Ogawa et al., 1990; Mukoyama et al., 1991) and their receptors (Fuller et al., 1988; Martin et al., 1989; Schulz et al., 1989). Additionally, proANP_1__–__9__8_ can break down into multiple peptides (Figure 1), i.e., amino acids 1–28 (proANP_*l*__–__28_), amino acids 31–67 (proANP_3__1__–__6__7_), and amino acids 79–98 (proANP_7__8__–__9__8_), which also have potent vasodilatory properties (Vesely et al., 1987). Interestingly, these proANP forms have been identified in the circulation in humans (Vesely, 1995). For instance, by using high performance-gel permeation chromatography (HPGPC) and radioimmunoassay (RAI), Vesely (1995) was able to demonstrate that proANP_1__–__9__8_ is further cleaved by proteases to generate these proANP fragments in the circulation. Among these forms, proANP_3__1__–__6__7_ has unique potent and prolonged diuretic and natriuretic properties (Gunning et al., 1992) and will be the main form described in the current review.

Here, we review in detail, the actions of the linear fragment proANP_3__1__–__6__7_, in particular on the heart, kidneys, and metabolism, which are independent of cGMP production. Originally, Gower et al. (1994) demonstrated the presence of different circulating molecular forms of the N-terminal and the C-terminal ANP prohormone peptides in plasma and their metabolites excreted in urine. These authors subjected plasma and urine samples from humans to high performance gel permeation chromatography (HP-GPC), followed by radioimmunoassay assessment of all ANP fragments, to reveal that proANP_3__1__–__6__7_ and ANP circulate as distinct peptides (Gower et al., 1994). Interestingly, the proANP_3__1__–__6__7_ levels in the circulation were found to be 10–20-fold higher than ANP_9__9–1__26_ in normal humans (Winters et al., 1989; Hartter et al., 2000) and dogs (Habibullah et al., 1995). This is explained by differences in the clearance rates of both peptides, i.e., it takes 45 min for proANP_3__1–6__7_ to be removed from the body compared to a half-life of 3–5 min for ANP (Greenwald et al., 1992). Additionally, proANP_3__1–6__7_ appears to be resistant to degradation by endopeptidases, such as neutral endopeptidase (NEP), being excreted in the urine largely intact (less terminal 2–3 a.a.) (Gower et al., 1994; Hartter et al., 2000). This unique characteristic of this polypeptide, contributes to the prolonged renal actions, and the potential therapeutic effects of proANP_3__1__–__6__7_.

It has been shown that the upstream and C-terminus fragments of the ANP prohormone are released by central hypervolemia induced by head-out water immersion (Vesely et al., 1989) or cardiac pacing or tachycardia (Ngo et al., 1989). These ANP fragments have physiologic actions similar to the ring structured ANP form, producing vasodilation (Vesely, 1995), natriuresis (Martin et al., 1990; Villarreal et al., 1999b), diuresis (Martin et al., 1990), and affecting metabolic phenotypes (Moro, 2013). In rodents, intravenous infusion of proANP_3__1__–__6__7_ (at doses of 0, 10, 30, and 100 ng/kg/min) in anesthetized normotensive and spontaneously hypertensive rats elicited natriuresis and diuresis (Villarreal et al., 1999b). Moreover, an increase in sodium excretion was also observed in intravenously infused anesthetized Munich-Wistar (Martin et al., 1990) and Sprague-Dawley (Dietz et al., 1994) rats. Both ANP and proANP_3l__–__67_ also inhibit sodium transport in suspensions of inner medullary collecting duct cells (Zeidel et al., 1986; Gunning et al., 1992). Because both peptides inhibit sodium transport in the collecting duct, it is possible that they act in an additive fashion on these cells in the intact animal. In conscious non-human primates (*Macaca fascicularis*), Benjamin and Peterson showed that infusion of proANP_3__1__–__6__7_ (15 pmol. kg^–*l*^. min^–*l*^ i.v.) increases renal sodium excretion, due to tubular and hemodynamic components (Benjamin and Peterson, 1995). Similarly, Vesely et al. (1994) demonstrated that intravenous infusion of proANP_3__1__–__6__7_ (100 ng/kg body weight/min, for 60 min) produced blood pressure-lowering, and diuretic and natriuretic properties in healthy individuals. Interestingly, these authors additionally showed that proANP_3__1__–__6__7_ has natriuretic properties that are significantly prolonged compared with ANP (Vesely et al., 1994).

NPs play also a key role in human metabolism (Cannone et al., 2019), thus connecting the heart with insulin−sensitive organs like adipose tissue, skeletal muscle, and liver. In fact, accumulation of NPs is associated with protein energy wasting and activation of browning in white adipose tissue (Luce et al., 2020). Importantly, ANP increases mitochondrial uncoupling and thermogenic gene expression in human adipocytes, induces thermogenic programs in brown (BAT) and white (WAT) adipose tissue and so increases energy expenditure (Bordicchia et al., 2012). These actions are considered favorable effects. However, in pathological conditions, these favorable actions are blunted or abolished. Accumulating evidence suggests that impaired cardiac endocrine function contributes to the development of obesity, type 2 diabetes, and other cardiometabolic complications (Verboven et al., 2017). The ring-structured form of ANP was reported to induce lipid mobilization and oxidation and to enhance insulin sensitivity (Coue and Moro, 2016). ANP infusion in humans increases energy expenditure and leads to lipolysis, with an increase in plasma levels of glycerol and non-esterified fatty acids regardless of body mass index (Birkenfeld et al., 2005, 2008). Also, intravenous administration of ANP increases plasma levels of adiponectin (Tsukamoto et al., 2009), an adipocyte-derived cytokine, which protects against atherosclerosis and insulin resistance or diabetes. However, the potential clinical utility of ANP might be limited by its inherent, sustained blood pressure lowering effects that can cause hypotension. Regarding the mid-sequence of the ANP, it has been shown that proANP_3__1__–__6__7_ might play an important role in the acute and chronic physiological responses to physical exercise. For instance, Cappellin et al. (2004) showed that measured proANP_3__1__–__6__7_ levels before and at the end of dynamic exercise in 28 trained cyclists and found that a single bout of exercise induce an increase in the urinary proANP_3__1–6__7_ levels. This could be, at least in part, explained by the increase in the venous return to the heart, and perhaps the higher heart rate levels, during a single exercise session. Interestingly, Freund et al. (1988) have demonstrated that the increase in the ANP levels occurs in a dose- and time-dependent manner. Additionally, proANP_3__1–6__7_ plasma concentration was also found higher in endurance trained athletes than in sedentary subjects (De Palo et al., 2000). Although protection of the vasculature, heart, and kidneys are favorable effects in the setting of metabolic diseases, the role of proANP_3__1__–__6__7_ in metabolism is unknown.

### Novel Therapeutic Strategies to Target ANP_3__1__–__6__7_

One of the hallmarks of heart failure (HF) is the marked increase in plasma levels of NPs (Burnett et al., 1986; Sugawara et al., 1988; Yamamoto et al., 1996; Cataliotti et al., 2001; Maisel et al., 2003; Richards and Troughton, 2004). It is established that elevated cardiac filling pressure is accompanied by increased circulating levels of ANP, and that congestive HF is not characterized by a deficiency in ANP, but with its elevation (Burnett et al., 1986). The increased circulating NP levels during HF are a compensatory response to volume overload and to hyperactivation of the adrenergic system and renin-angiotensin-aldosterone system (RAAS). However, not all HF patients seem to increase the circulating levels of NPs. In a recent clinical study, approximately 26% of acutely decompensated heart failure patients presented a lack of increase of circulating levels of ANP (Reginauld et al., 2019), which might suggest the existence of a relative state of ANP deficiency in a subgroup of patients, possibly due to reduced production, altered release, or enhanced enzymatic degradation by neprilysin. It should also be underscored, however, that the role of potential confounders responsible for this apparent ANP deficiency status remains yet to be fully elucidated (Richards and Januzzi, 2019). Nevertheless, as discussed herein, the increased cardiac production and circulation of NPs can be differently processed in the periphery in chronic HF patients, resulting in inactive forms with no efficient benefit, thus supporting the rational for using NPs or their analogs as anti-HF therapy (Belluardo et al., 2006; Macheret et al., 2012). Others and we have previously demonstrated the existence of a deficiency state of the endogenous biologically active NPs system in HF patients starting with the early stage of HF (Hawkridge et al., 2005; Belluardo et al., 2006; Niederkofler et al., 2008; Macheret et al., 2012). Additionally, a blunted natriuretic response has been observed after treatment with different pharmacological agents (e.g., angiotensin-converting enzyme inhibitors, angiotensin-II blockers, β-blockers, and spironolactone) in experimental models and in patients with chronic heart failure, suggesting a resistance to the biological effects of NPs (Cody et al., 1986; Saito et al., 1987; Komeichi et al., 1995; Charloux et al., 2003). This resistance to biological effects of ANP is probably mainly due to up-regulation of clearance receptors in patients with chronic heart failure (Andreassi et al., 2001; Clerico et al., 2006).

Winters et al. (1989) have evaluated the N-terminus and C-terminus ANP fragments in the circulation of thirty patients with varying severity degrees of congestive HF using high-pressure liquid chromatography. Compared to the other ANP peptides, proANP_3__1__–__6__7_ was the only one that accurately discriminated the severity of congestive HF (Winters et al., 1989). In light of these findings, the impaired production and release of mature forms of the NPs and of their linear precursors seems to play a fundamental role in the evolution and progression of HF, and thus the exogenous supplementation of such cardiac hormones may prove to be of therapeutic importance in HF. In fact, the biologic properties of the NPs have supported the development of as therapeutic agents for cardiovascular diseases (Marcus et al., 1996; Yamamoto et al., 1997; Colucci et al., 2000; Hobbs et al., 2001; Boerrigter and Burnett, 2004; Rubattu et al., 2019; Rubattu and Volpe, 2019). Here we will further discuss the development of novel therapeutic strategies based on exogenous supplementation of a linear fragment of the ANP, the proANP_3__1__–__6__7_, in HF.

The effort to develop novel therapeutic strategies to prevent the progression of cardiovascular disease is also focused on restoring the impaired NP system, for instance, by augmenting the circulating levels of NPs through exogenous supplementation. For instance, the NP drugs carperitide and nesiritide have been approved for use in patients in Japan and United States, respectively, as intravenous agents for the treatment of acute decompensated HF. These forms stimulate the production of cGMP, and frequently result in inadequate cardioprotective effects due to significant reductions in blood pressure levels, leading to reduced renal perfusion and further deterioration of kidney function.

ProANP_31__–__67_ was shown to enhance renal function acutely in persons with congestive heart failure (Vesely et al., 2001) and to protect against ischemia-induced acute tubular necrosis and renal failure in a rat model of ischemic non-oliguric acute renal failure (Clark et al., 2000). Overall, proANP_31__–__67_ induced renal vasodilation and diuresis with enhanced sodium excretion, but with no associated increase in oxygen consumption. Of note, as mentioned above, the biological actions of proANP_3__1__–__6__7_ were independent of cGMP activation and therefore, are characterized by a less intense vasodilatory effect (Vesely et al., 1987).

Potential clinical indications for proANP_3__1__–__6__7_ include reducing symptoms in patients with worsening HF or those diagnosed with stable congestive HF and compromised renal function, or cardiorenal syndrome. Clinical trials showing safety and efficacy of synthetic proANP_31__–__67_ peptide were conducted on stable congestive HF and renal impairment patients (ACTRN12612000576820 and ACTRN12611000806965), and on acute decompensated congestive HF patients (ACTRN12609000998246). Intravenous and subcutaneous delivery of proANP_3__1__–__6__7_ was shown to preserve renal function in both chronic and acute heart failure with reduced ejection fraction (HFrEF) (Delacroix et al., 2016). Additionally, the infusion of proANP_3__1__–__6__7_ (100 ng/kg/min, i.v. for 1 h) has been shown to possess several cardiac enhancing effects in congestive HF patients (NYHA III), including augmenting cardiac output, cardiac index, and stroke volume index, while reducing pulmonary capillary wedge pressure (Vesely et al., 1998). Of note, proANP_3__1__–__6__7_ has similar effects to those observed with the ring forms of the NPs, which are currently in use for the treatment of acute decompensated overt HF, but has shown a less intense blood pressure lowering effect.

Based on these observations and on the known unique renal protective effects of proANP_31__–__67_, we investigated the therapeutic value of proANP_31__–__67_ for maladaptive cardiac and renal remodeling in a rat experimental model of salt-induced hypertension (Altara et al., 2020). This is a preclinical model for heart failure with preserved ejection fraction (HFpEF), as evidenced by concentric remodeling/hypertrophy and diastolic dysfunction, i.e., increased cardiac stiffness. We also sought to extend current knowledge on the protective actions of chronic exogenous supplementation of proANP_31__–__67_ on the kidney, knowing that it stimulates natriuresis and diuresis, but has moderate effect on blood pressure. With hypertension in this preclinical model, we observed that proANP_31__–__67_ increased urine output, natriuresis, and glomerular filtration rate (GFR), while preventing detrimental perivascular collagen deposition in the renal cortex. Remarkably, proANP_31__–__67_ was shown to be beneficial to the heart. Characteristic signs of adverse cardiac remodeling and function that manifested as diastolic dysfunction were attenuated with chronic administration of proANP_31__–__67_. These beneficial actions on the heart, included attenuated cardiac hypertrophy, as indicated by decreased heart weight to body weight ratio and left atrial diameter, as well as reduced fibrosis (both interstitial and perivascular left ventricular fibrosis) and normalized ratio of the diastolic mitral inflow E wave to A wave, a measure of cardiac stiffness (Altara et al., 2020). Of note, the beneficial effects on the heart were retained absent of a marked lowering of blood pressure and when animals were treated with a renal sub-therapeutic dose of proANP_31__–__67_, suggesting a unique mode of action directly on the heart, beyond its renal actions, which warrants further investigation.

## Mechanisms of Action of Anp_3__1__–__6__7_

The mechanisms of action of proANP_3__1__–__6__7_ associated with the cardiorenal protective and diuretic effects were not attributed to any effects on blood pressure. Competitive binding experiments revealed that proANP_3__1__–__6__7_ does not activate the canonical NP receptors (e.g., NPR-A and NPR-B), resulting in the activation of the cGMP pathway but rather has its own separate and distinct receptor (Vesely et al., 1990, 1992). However, the nature of the proANP_3__1__–__6__7_ receptor is still unknown.

### Renal Mechanisms

**FIGURE 2:**
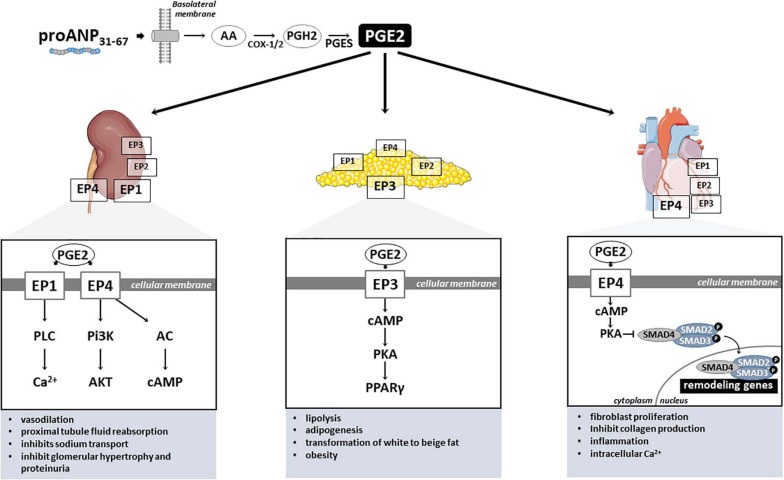
PGE_2_-mediated proANP_3__1__–__6__7_ cardiac, renal and metabolic mechanisms of action. PGE_2_, Prostaglandin E2; EP1–4, PGE_2_ receptors (coupled to G_*q*_); AA, Arachidonic acid; COX-1/2, Cyclooxygenase-1 and –2; PGH2, Prostaglandin H2; PGES, Prostaglandin E synthase; PLC, Phospholipase C; Pi3K, Phosphoinositide 3-kinases; AC, Adenylate cyclase; Ca^2+^, Calcium ion; AKT, protein kinase B; cAMP, Adenosine 3′,5′-cyclic monophosphate; PKA, protein kinase A; SMAD2–4, SMAD (Mothers against decapentaplegic) family member 2, 3, and 4; PPARγ, Peroxisome proliferator-activated receptor gamma.

With regard to its mechanism of action in the kidney, several studies have shown that proANP_3__1__–__6__7_ endogenously (i.e., paracrine like) induces prostaglandin E_2_ (PGE_2_) formation (Gunning et al., 1992; Vesely et al., 2000). PGE_2_, a product of the cyclooxygenase 2 (COX-2) pathway (Figure 2), is an important homeostatic regulator of nephropathy, as well as hypertension, adipogenesis, dyslipidemia, diabetes, neuropathy, atherogenesis and retinopathy, contributing to global cardiovascular risk (Nasrallah et al., 2016). PGE_2_ was also reported to modulate growth, fibrosis, and apoptosis phenotypes by influencing inflammatory, immune, and oxidative stress responses (Makino et al., 2002; Zahner et al., 2009; Nasrallah et al., 2015). Felder et al. (2017) reported that proANP_3__1__–__6__7_ directly stimulated PGE_2_ release in the renal medullary tissue, more precisely by cells located in the collecting tubules/ducts at the cortical-medullary interface. Through PGE_2_, proANP_3__1__–__6__7_ is also a potent inhibitor of the Na^+^-K^+^-ATPase (or sodium-potassium pump) of inner medullary collecting duct cells, resulting in Na^+^ transport inhibition and natriuretic actions. Furthermore, proANP_3__1__–__6__7_ was shown to increase GFR, both in preclinical models as well as in humans with congestive HF, and to attenuate tubular necrosis in a rat model of acute renal failure (Afsar et al., 2017). Intrarenal administration of proANP_3__1__–__6__7_ also increases creatinine clearance and inhibits renin secretion in a Na^+^ depleted canine model of renin system activation induced by unilateral nephrectomy (Villarreal et al., 1999a). These data suggest that inhibition of renin secretion is, at least in part, in response to a proANP_3__1__–__6__7_-induced increase in the sodium load delivered to the macula densa.

In mammals, PGE_2_ exerts its signals through four G protein-coupled receptors, designated EP1, EP2, EP3, and EP4 (Figure 2; Sugimoto and Narumiya, 2007). Although highly conserved among mammals, the PGE_2_ receptors have distinct signal transduction pathways, and tissue and cellular distribution, reflecting their diverse properties (Nasrallah et al., 2016). In the kidney, EP1 and EP4 receptors seem to mediate PGE_2_ microcirculation actions (Figure 2). Purdy and Arendshorst (2000) identified by RT-PCR the expression of EP1 (*Ptger1*) and EP4 (*Ptger4*) receptors in freshly isolated preglomerular arterioles of Sprague-Dawley rats. These authors also demonstrate that the EP4 receptor is the major receptor located in preglomerular vascular smooth muscle cells, mediating PGE_2_-induced vasodilation through cAMP formation and reduction of cytosolic Ca^2+^ levels (Purdy and Arendshorst, 2000). Curiously, the renal vascular tone response induced by PGE_2_ stimulation seems to vary depending on the type of the receptor (Schweda et al., 2004). For instance, EP2^–/–^ and EP4^–/–^ mice presented an augmented vasoconstriction in response to higher PGE_2_ concentrations, contrasting with the markedly blunted response observed in EP1 and EP3 knockout mice (Schweda et al., 2004). Furthermore, EP1 and EP4 were detected in transformed murine proximal tubular cells (MCTs), mediating PGE_2_-induce fluid reabsorption (Nasrallah et al., 2015). Loss-of-function *in vivo* experiments in mice have shown that PGE_2_ stimulates the renin-angiotensin-aldosterone system by activation of EP4 receptor (Poschke et al., 2012). Similarly, Schweda et al. (2004) demonstrated that PGE_2_ stimulates renin release in juxtaglomerular cells via activation of both EP2 and EP4 receptors. Interestingly, the EP1 receptor attenuates vasopressin-dependent water reabsorption and inhibits sodium transport in the collecting duct (Nasrallah et al., 2018). Activation of PGE_2_-EP4 signaling with proANP_3__1__–__6__7_ also can exert multiple biochemical effects on the kidney and other organs, suggesting the potential wide-ranging use of EP4 in both cardiovascular and metabolic disorders. For instance, by inhibiting Na^+^ transport in the inner medullary collecting duct cells, proANP_3__1__–__6__7_ is known to reduce renal oxygen consumption (Gunning et al., 1992).

Under different pathological conditions, the PGE_2_ receptors seem to be involved in the development of renal disease. For instance, the oral administration of PGE_2_ receptor EP1-selective antagonist prevented the progression of nephropathy, evidenced by improved glomerular hypertrophy, decreased mesangial expansion, and suppression of proteinuria in streptozotocin-induced diabetic rats (Makino et al., 2002). Mechanistically, the authors demonstrated that mesangial cells cultured under high-glucose conditions and treated with this selective agonist for EP1 receptor exhibit inhibited transforming growth factor-beta (TGF-β) and fibronectin upregulation, key regulators of the extracellular matrix. Similarly, an EP4-specific agonist significantly attenuated the development of tubulointerstitial fibrosis induced by unilateral ureteral obstruction in mice by suppressing inflammatory responses (Nakagawa et al., 2012). On the other hand, knockout mice for EP4 showed exacerbated tubulointerstitial fibrosis response after ureteral obstruction. Additionally, cultured renal fibroblasts treated with EP4 agonist significantly inhibited the platelet-derived growth factor (PDGF)-induced proliferation and profibrotic connective tissue growth factor production (Nakagawa et al., 2012). Hence, these data indicate that both PGE_2_ receptors EP1 and EP4 play critical roles in the development of renal injury (Figure 2), and might explain the renal protective benefits of proANP_3__1__–__6__7_ observed by our group in hypertensive rats. For instance, chronic administration of proANP_3__1__–__6__7_ prevented perivascular collagen deposition in the rat experimental model of salt-induced hypertension, accompanied by improvements in renal function (Altara et al., 2020).

### Myocardial Mechanisms

Cardiac phenotypes are equally affected by the different PGE_2_ receptors (Figure 2). Although all four PGE_2_ receptors are detected in the cardiac tissue, EP4 is highly expressed in the heart (Muraoka et al., 2019), and seems to have protective effects against adverse remodeling. In fact, EP4 agonist administration to mice subjected to pressure overload (Wang et al., 2017) and cardiac injury (Hishikari et al., 2009; Pang et al., 2016) exhibited antifibrotic effects and prevented the progression to systolic dysfunction. In a mouse model of cardiac hypertrophy generated by transverse aortic constriction (TAC) surgical procedure, EP4 agonist ONO-0260164 treatment significantly prevented myocardial fibrosis and progression of systolic dysfunction 5 weeks after pressure overload (Wang et al., 2017). Hishikari et al. (2009) used another EP4 selective agonist (EP4RAG) to treat rats submitted to myocardial ischemia-reperfusion injury and demonstrated that EP4RAG significantly reduced ischemic myocardium, attenuated interstitial fibrosis, and ameliorated cardiac contractility and dilatation compared with vehicle.

The generation of genetically engineered animals has contributed with the understanding of the role of PGE_2_ receptors in the cardiac tissue. Qian et al. (2008) generated cardiac specific EP4 deficiency, using site-specific recombination by the Cre recombinase method (Cre-loxP) to inactivate EP4 only in cardiomyocytes (CM- EP4 knockout [KO]), and showed that CM-EP4 KO mice are defective in their ability to activate Stat-3, presenting a worsening of systolic function after myocardial infarction injury. These studies are interpreted as indicating that EP4 plays both protective and damaging roles in the heart with the protective effects of EP4 due at least in part to its ability to suppress inflammation.

We cannot exclude, however, the role of PGE_2_ stimulation of its receptors in cells other than cardiomyocytes, for instance cardiac fibroblasts, endothelial cells, and smooth muscle cells. In fact, it has been demonstrated recently that EP4 signal also regulates fibrotic phenotypes in cardiac fibroblasts. In this regards, Umemura et al. (2019) showed that cardiac fibroblasts isolated from adult rats treated with EP4 agonist (ONO-AE1-437) decreased the expression of transforming growth factor-β (TGF-β), connective tissue growth factor (CTGF) and ACTA2 (a-smooth muscle actin) mRNA, suggesting that that EP4 signaling suppresses fibroblasts to myofibroblast transdifferentiation. Consistently, Wang et al. (2017) demonstrated in cultured neonatal rat cardiac fibroblasts that treatment with EP4 agonist ONO-0260164 inhibited the TGF-β1 induced upregulation of collagen type 1 (*Col1a1*) and type 3 (*Col3a1*) gene expression.

Mechanistically, EP4 is a G protein-coupled receptor with seven transmembrane domains that when bound to PGE_2_ or another agonists, mobilizes G proteins containing the Gs alpha subunit (i.e., Gαs) and G beta-gamma (i.e., Gβγ) (Tuteja, 2009). In particular, Gsα stimulates adenylyl cyclase to raise the production of cyclic adenosine monophosphate (cAMP) (Yokoyama et al., 2013), that subsequently activates protein kinase A (PKA), which in turn phosphorylates downstream proteins, such as the transcription factor cAMP response element binding protein (CREB). Of note, CREB regulates the expression of genes that control cellular proliferation, cellular differentiation, cellular survival, and angiogenesis. The activated CREB(p) binds to specific sites and regulates the expression of genes, such as B-cell lymphoma 2 and tumor necrosis factor α (TNFα), which are involved in development of ischemic heart disease (Ichiki, 2006). EP4 activation of G proteins also triggers PI3K/AKT/mTOR, ERK, and p38 MAPK pathways (Xu et al., 2018). Regarding the other PGE_2_ receptors expressed in the cardiac tissue, it is known that PGE_2_ stimulates cardiac fibroblast proliferation via both EP1 and EP3, p42/44 MAPK and Akt-regulation of cyclin D3, possibly modulating cardiac fibrosis (Harding and LaPointe, 2011).

As mentioned, in addition to its renoprotective effects, proANP_3__1__–__6__7_ inhibited cardiac hypertrophy and early onset of diastolic dysfunction in our salt-induced hypertension model of HFpEF, as indicated by reduced cardiac fibrosis (Altara et al., 2020). The cardioprotective actions of proANP_3__1__–__6__7_ may have resulted from a local increase in PGE_2_ and activation of the EP4, which recently has been demonstrated to have antifibrotic and antihypertrophic actions in the heart (Yamagami et al., 2015; Harada et al., 2017; Wang et al., 2017; Bryson et al., 2018; Lai et al., 2018; Zhu et al., 2019; Jin et al., 2020). ProANP_3__1__–__6__7_ seems to activate the PGE_2_-EP4-SMAD signaling pathway, reducing the phosphorylation of SMAD2 (Altara et al., 2020), possibly inhibiting the activation in TGF-β1 mediated collagen deposition. Evidence indicates that EP4 attenuates cardiac fibrosis by inhibiting SMAD signaling through activation of protein kinase A (PKA) (Harada et al., 2017; Wang et al., 2017). In our study, urine levels of PGE_2_ were elevated by proANP_3__1__–__6__7_, although we did not observe a significant increase in plasma PGE_2_. However, local PGE_2_ production in the heart, where levels tended to be increased by treatment with proANP_3__1__–__6__7_, may have been responsible for the inhibition of cardiac remodeling process observed in our study (Altara et al., 2020), Therefore, the cardioprotective actions of proANP_3__1__–__6__7_ observed in our study (e.g., improved diastolic function, attenuated cardiac fibrosis and hypertrophy, and anti-remodeling effect on cardiomyocytes) (Altara et al., 2020) may have resulted from the activation of the EP4.

However, this response might be dependent on the cardiac cell type involved. In fact, proANP_31__–__67_ may have direct effects on the ultrastructure of cardiomyocytes. We have demonstrated that chronic administration of proANP_3__1__–__6__7_ reduced t-tubule density in our rat model of hypertensive heart disease and renal damage (Altara et al., 2020). Normal ultrastructure of cardiac t-tubules is important in electrical-mechanical coupling and Ca^2+^ handling in cardiomyocytes as any abnormalities may predispose toward heart failure (Manfra et al., 2017). In HF patients, we have demonstrated etiology-dependent differences in mechanisms for diastolic dysfunction (Frisk et al., 2021). For instance, myocardial biopsies from HFrEF hearts under high ventricular wall stress were linked to disruption of t-tubules, local collagen deposition and of systolic calcium homeostasis impairment. In contrast, maintained wall stress in HFpEF patients was associated with compensatory t-tubule proliferation and largely maintained calcium release (Frisk et al., 2021). In keeping with this, we observed that proANP_3__1__–__6__7_ treatment protects t-tubular structure and density, and also preserves intracellular distances between t-tubules and the sarcolemmal membrane. In our study, we did not examine the effect of proANP_3__1__–__6__7_ on Ca^2+^ dynamics in cardiomyocytes, which remains an area for future investigation.

Taken together, our study previously discussed supports the hypothesis that proANP_3__1__–__6__7_ cardioprotective benefits might directly affect both cardiac fibroblasts and cardiomyocytes, via the activation of PGE_2_/EP4 signaling. We cannot exclude the possibility that proANP_3__1__–__6__7_ mediates the secretion of growth factors by myofibroblasts indirectly induces hypertrophy of cardiomyocytes via a paracrine like-manner, which is a landmark of heart failure. Therefore, the detailed investigation of the proANP_3__1__–__6__7_ molecular mechanisms involving anti-fibrotic signaling pathways and cellular processes, including inflammation, signaling kinases, apoptosis, fibroblast-to-myofibroblast differentiation, cardiomyocytes ultrastructure is absolutely crucial to understand the cardiorenal protective actions of this compound in a heart failure scenario.

### Mechanisms Associated With Metabolic Phenotypes

With regard to the role of PGE_2_ mediating metabolic phenotypes, these effects seem to be mainly mediated by EP3 and EP4 receptors in the adipose tissue. Of those, the EP3 is the most widely abundant receptor in adipose tissue (Tang et al., 2015; Xu et al., 2016), and is involved in various pathophysiological processes (Cai et al., 2015). Accordingly, PGE_2_ receptor EP3 seems to regulate both lipolysis and adipogenesis in white adipose tissue (Strong et al., 1992; Fain et al., 2000; Xu et al., 2016), as well as adipocyte transformation of white to beige fat, protecting against obesity and metabolic disease (Garcia-Alonso et al., 2013). In fact, it has been shown that loss-of-function of EP3 in mice resulted in obese and insulin resistant phenotypes (Sanchez-Alavez et al., 2007; Ceddia et al., 2016). Mechanistically, using both pharmacological blockade and genetic disruption, Xu et al. (2016) elegantly showed that PGE_2_ EP3 receptor inhibits adipogenesis via the cAMP/PKA/PPARγ pathway, and blocks lipolysis mainly through the cAMP/PKA/HSL pathway in white adipose tissue. These data demonstrates that PGE_2_/EP3 axis is critical for lipid and glucose metabolism.

Activation of PGE_2_-EP4 signaling seems also to exert important role in adipose tissue and metabolic disorders. Loss-of-function mice model for EP4 submitted to high fat diet exhibited reduced body weight gain and adiposity, and shorter life span when compared with wild type (Cai et al., 2015). Additionally, EP4 deficiency induced disruption in lipid metabolism due to impaired triglyceride clearance (Cai et al., 2015). Nevertheless, it is still unknown any direct or indirect metabolic properties of proANP_3__1__–__6__7_ and future investigation is fundamental. However, there are some evidence of the possible connection of proANP_3__1__–__6__7_ and metabolic phenotypes. For instance, in inner medullary collecting duct (IMCD) cells it has been previous shown that proANP_3__1__–__6__7_ reduces O_2_ not by direct inhibition of mitochondrial O_2_ consumption, but by reducing the demand for metabolic energy of the Na^+^-K^+^-ATPase (Gunning et al., 1992). Accumulation of NPs is, in fact, associated with protein energy wasting and activation of browning in white adipose tissue (Luce et al., 2020). The incubation of primary adipose cells exposed to ANP led to a significant increase of uncoupling protein 1 content. Therefore, it is reasonable to believe that proANP_3__1__–__6__7_ might also has potential in metabolic disorders associated or not with cardiovascular diseases.

## Potential Useful Combination of proAnp_3__1__–__6__7_ With Current Medications

Notwithstanding the substantial advance achieved in treatment, the incidence of heart failure has not been reduced and remains the major cause of morbidity and mortality in developing and developed countries (Roger, 2013). Nevertheless, current medical procedures aim to increase survival of cardiac tissue and limit cardiac damage, whereas an effective treatment to improve and/or protect renal function, which often deteriorates after cardiac injury, is still lacking and urgently needed. More recently, the combination of NEP inhibitor (Sacubitril) and angiotensin II receptor blocker (Valsartan) (sold as Entresto) became a first-choice treatment for HFrEF patients (Volpe et al., 2015; Seferovic et al., 2019; Volpe et al., 2019), based on its superior benefits to reduce cardiovascular death, and HF symptoms and hospitalizations compared to angiotensin-converting enzyme inhibitor (ACEi) (McMurray et al., 2014). Inhibition of NEP, by decreasing NPs degradation (Zile et al., 2016), elicits hypotensive actions. Of note, Entresto presented higher proportions of hypotension and non-serious angioedema cases, but lower proportions of patients with renal impairment, hyperkalemia, and cough than ACEi (Enalapril). In this regards, proANP_3__1__–__6__7_ may provide an ideal complementary therapeutic strategy by directly targeting end-organ remodeling in the setting of HFpEF. Given the fact that proANP_3__1__–__6__7_ does not appreciably lower blood pressure, this peptide may be especially efficacious as an add-on-therapy to target end organ damage, and could be tested in other heart disease settings, including coronary heart disease, as well as aortic valve stenosis, cardiac dysfunction in the presence of metabolic disease, along with both forms of HF (e.g., HFpEF or HFrEF), and cardiorenal syndrome, for which it has been first developed. An additional beneficial action of proANP_3__1__–__6__7_ is increased urinary excretion of potassium, suggesting that this peptide might be combined with potassium sparing drugs or medications frequently used in the treatment of HF (Altara et al., 2020). An interesting area of future investigation would be the impact of proANP_3__1–6__7_ on the metabolic syndrome, a collection of conditions that contribute to the development of heart disease, diabetes and stroke. We anticipate a synergistic beneficial action of proANP_3__1__–__6__7_ in preventing end organ damage and attenuating metabolic dysfunction when used in combination with current therapies to ameliorate lipid and glucose metabolism. Of note, proANP_3__1__–__6__7_ pharmacokinetics and high stability also render it an interesting candidate as therapeutic agent for chronic administration. Equally important, no adverse effects, gross or microscopic pathology changes were observed when proANP_3__1__–__6__7_ was tested in various pre-clinical models (Benjamin and Peterson, 1995; Villarreal et al., 1999b; Clark et al., 2000; Vesely, 2007; Altara et al., 2020), ranging from mice to non-human primates (monkeys or Macaca fascicularis). With respect to the administration to patients, proANP_3__1__–__6__7_ proved to be well tolerated at all doses via both intravenous (IV) and subcutaneous (SQ) infusions, without the profound vasodilatory and hypotensive complications evident with CT ring agents (blood pressure was maintained with no adverse hemodynamic effects noted) (Vesely et al., 1994, 1998, 2000). Additionally, pharmacokinetic analysis with pharmacodynamic parameters showed no adverse effects on any parameters measured, including cardiac and renal performance.

## Conclusion

Considering that unique mechanism of action, its intrinsic resistance to enzymatic degradation, and its complementary actions to other members of the NPs system, make it compelling to evaluate the effects of proANP_3__1__–__6__7_, as single therapy as well as in combination with current medications, in the treatment of cardiac diseases and metabolic syndrome. The long half-life, and its safe pharmacological profile, make proANP_3__1__–__6__7_ a promising therapeutic option for currently difficult to treat clinical conditions. Therefore, further studies aimed to demonstrate its protective effects and exploit its clinical potential are clearly warranted.

## Author Contributions

AC was the lead author and conceived the concept of the manuscript. All authors contributed to the literature review and writing of the manuscript and approved the final version.

## Conflict of Interest

AC serves as consultant for the advisory board of Madeleine Pharmaceuticals. RA and AC have applied for a patent related to the cardiac protective properties of proANP_31–67_ (UK # 2000945.2). The remaining authors declare that the research was conducted in the absence of any commercial or financial relationships that could be construed as a potential conflict of interest.
